# Prevalence of damaged and missing teeth among women in the southern plains of Nepal: Findings of a simplified assessment tool

**DOI:** 10.1371/journal.pone.0225192

**Published:** 2019-12-03

**Authors:** Priyanka Agrawal, Swetha Manohar, Andrew L. Thorne-Lyman, K. C. Angela, Binod Shrestha, Rolf D. Klemm, Keith P. West

**Affiliations:** 1 International Injury Research Unit, Department of International Health, Johns Hopkins Bloomberg School of Public Health, Baltimore, Maryland, United States of America; 2 Center for Human Nutrition, Department of International Health, Johns Hopkins Bloomberg School of Public Health, Baltimore, Maryland, United States of America; 3 Center for Human Nutrition, Department of International Health, Johns Hopkins Bloomberg School of Public Health, Baltimore, Maryland, United States of America; 4 Department of Nutrition, Harvard T.H. Chan School of Public Health, Boston, Massachusetts, United States of America; 5 PoSHAN Study Team, Johns Hopkins University, Kathmandu, Nepal; 6 Center for Human Nutrition, Department of International Health, Johns Hopkins Bloomberg School of Public Health, Baltimore, Maryland, United States of America; 7 Helen Keller International, New York, New York, United States of America; Centre Hospitalier Regional Universitaire de Tours, FRANCE

## Abstract

**Objective:**

To assess the prevalence of missing and damaged teeth among women in the rural southern plains of Nepal using an interviewer-administered tooth assessment module.

**Setting:**

21wards in seven Village Development Committees across the Tarai of Nepal in 2015.

**Participants:**

Resident, married women of children less than 5 years of age or those married in the 2 years prior to the survey, 14 to 49 years of age participating in a mid-year nutrition and health survey in the Tarai region of Nepal.

**Outcome measures:**

Prevalence of missing and damaged teeth, history of dental problems, oral hygiene practices, access to dental treatment and risk factors for missing and damaged teeth.

**Results:**

Of 3007 assessed women, aged 14 to 49 years of age, 22.8% (95% CI: 21.4–24.4) reported ≥ 1 missing or damaged teeth; 81.5% (95% CI 80.1–82.9) reported regularly practicing oral hygiene, typically with standard local dentifrices. Pain or discomfort in the oral cavity in the previous 6 months affected 17.6% of women. Among these, 43.8% had sought treatment from a dental facility, pharmacy or village doctor. Home remedies were commonly applied to relieve pain.

**Conclusion:**

Broken and missing teeth are common, affecting nearly one-quarter of adult women of reproductive age in rural Southern Nepal, as assessed by an interviewer-administered questionnaire.

## Introduction

Oral health complications are poorly documented in most low- and middle-income countries (LMICs). Reliable prevalence estimates, with consequent quality of life and economic burdens, of untreated dental diseases, remain virtually absent to date for lack of technical capabilities in resource poor settings [1, 2]. At present, only 38% of all LMICs have a national oral health data set.[3] Rising consumption of sweet foods and sugary drinks, characteristic of a nutrition transition toward obesity that is underway in most LMICs coupled with insufficient oral health education programs, school-based interventions and primary oral health care and prevention strategies, have been cited as reasons for the high prevalence of dental decay and related pain in developing countries.[1, 4] Notwithstanding the paucity of data, estimates that do exist suggest poor dental health to be a global public health problem, with 3.5 billion people having treatable, morbid dental conditions [5]

A challenge to revealing the burden of dental disease and monitoring the prevention of decay through improved care stems from a lack of practical and reliable oral health diagnostics by front line community health workers and lay, trained surveyors. Commonly dental disease prevalence measures and indices such as the World Health Organization’s (WHO) recommended “decayed, missing, filled teeth” index (DMFT), the Oral Hygiene Index—Simplified (OHI-S), and the Community Periodontal Index for Treatment Needs (CPITN) continue to remain under the sole purview of dental health scientists, clinicians and technical specialists, [6] although structured questionnaires have occasionally been used to gather information on dental decay and pain by trained dentists in school and village settings. [1, 2, 7, 8]. The comprehensive Global Burden of Disease (GBD) study highlights additional challenges [5]. Specifically, while generating estimates of the burdens of most severe oral diseases, the GBD fails to capture antecedent conditions to severe periodontitis such as early tooth decay or poor oral hygiene practices that precede clinical disease [5] Current survey systems are expensive, time consuming, overly-reliant on technology and, thus, often unable to reliably function under usual field conditions in underdeveloped settings. A simple, oral health assessment tool presented here overcomes common logistical and technical challenges in the field while capturing credible data on oral disease, injury manifested by tooth damage or loss and oral hygiene practices among women of reproductive age. The assessment was nested into an agriculture, nutrition and health survey in the rural southern plains (Tarai) of Nepal.

## Methods

A tooth and oral health practices module was embedded in the Policy and Science for Health, Agriculture and Nutrition (PoSHAN) Community Studies project in Nepal, comprising a previously described series of nationally representative, annual cross-sectional surveys carried out from 2013 to 2016.[9] The PoSHAN surveys were designed to assess the extent, direction and strengths of association between agricultural practices, markets, extension programs, and home food security and maternal and preschool child diet, nutrition and health in Nepal that may inform and guide policies and programs. [9]

Eligibility criteria for accessing women in the surveys included (1) presence of a child under-5 years of age or (2) being without a young child but married within the past 2 years. As risk of missing and damaged teeth is known to correlate with age, the present analysis focused on women in our sample between the ages of 14 and 49 years of age. The core survey tool included detailed information collected at the community, household, woman and child levels, including measures of local market food prices, agricultural productivity, food security, and diet, nutritional status and health of women and children, and exposure to agricultural and microeconomic extension, nutrition and health programs in Nepal. The surveys’ protocol also provided a platform to nest sub-studies to explore specific health burdens within the population. Thus, in 2015, a module was developed and fielded to collect data on oral health indicators in the form of a self-reported tooth assessment module.

Due to the large earthquake that struck the hill and mountain regions of Nepal in April-May 2015, mid-year data collection that year was limited to the least affected southern agroecological zone the Tarai, where seven Village Development Committees (VDCs) (Banke, Nawalparasi, Bara, Sarlahi, Dhanusa, Saptari, Morang districts) were systematically sampled from West to East following a random start. One VDC each in the mountains and hills were also assessed, but not addressed in this report (S1 Fig).[9–11] Thus, the present study focuses on generating representative estimates of tooth loss and damage in the Tarai zone, where ~60% of the population of Nepal resides.

All heads of households were interviewed to obtain information on demographic, household socioeconomic, food security and dietary characteristics. Eligible women were interviewed to collect data on history of pregnancy, pre- and post-natal care, dietary frequency over the previous 7 days, and morbidity history over a period of 30 days prior to the interview, access to health and nutritional services within a year prior to the interview, and knowledge and practices related to maternal health and nutrition, anthropometry (weight, height and left mid-upper arm circumference of women) and hemoglobin measurements in a random 10% subset of women in selected households, obtained by finger sticks and measurement on an HB 201 hemoglobin meter (HemoCue AB, Angelholm, Sweden).

The tooth assessment module (available in S1 File) collected information on histories of dental problems, oral hygiene practices, self-reported history of dental pain (recall period of 6 months), and access to treatment for dental complaints. The main outcome of study was the self-reported counts of missing and/or damaged teeth among mothers or female caretakers of children under 5 years, and recently married women without children, aided by a pictorial diagrammatic simulation of the oral cavity. (S2 Fig)

A missing tooth was defined as “any tooth in the oral cavity lost due to dental extraction, natural causes, or traumatic injury”. Damaged teeth were defined as “any carious and grossly damaged tooth, root stumps, cracked or chipped teeth, abrasions, and mottled teeth.”

Prior to field work, a training session consisting of a day-long simulation exercise was conducted for a team of 21 master trainers who also served as enumerators, none of whom had ever worked in oral health. Since the module had initially been developed in English and translated into Nepali, training included exercises in back translation to assure unambiguous translation, and practice exercises to ensure consistent performance. The team was also oriented with dental terminology used in the assessment module, structure of the human mouth, and mechanics of identifying onset of caries. These master trainers then trained the remaining team of 42 enumerators on the tooth assessment module. During the survey, the data collectors walked eligible women through each question in the module and recorded responses. The enumerators assisted the women in conceptualizing the pictorial diagram and orienting it with their own oral cavity to be able to report the number of missing and/or damaged teeth in their mouth. The data collected was regularly checked in the field for outliers, consistency and completeness. It took approximately 10 minutes to complete the tooth assessment module and no significant difficulties were reported from enumerators.

The sampling approach was designed under the assumption that it would accrue approximately 2500 women in the Tarai, and with this sample size, for a prevalence of approximately 20% (a reasonable prior approximation of prevalence of damaged teeth), we had power (1-β) = 0.97 to report a prevalence.

Descriptive statistics provided estimates of frequency dsitributions for missing and damaged teeth by socio-demographic factors such as age, occupation, educational level, nutritional status, food security, and diet history. A wealth index was calculated as a measure of socioeconomic status using principal component analysis of data on household asset and construction materials, including electricity, source of energy for cooking, source of drinking water, type of toilet facility, and availability of cooking fuel, main material of wall, roof and floor, and household asset ownership, previously described.[12] The wealth index ranged from -5.0 to 5.2, but was converted to a binary variable with values <0 and ≥ 0 for purposes of analysis. Women’s intake of different food items was measured using the 51-item 7-day food frequency questionnaire, adapted from previous studies conducted in Nepal. [13] The aim of including dietary data in the present analysis was to evaluate possible adverse effects of poor dental health on usual intake. Food items were consolidated into nine food groups, based on the Food and Agricultural Organization’s Household Dietary Diversity Score guidance, including starchy staples; legumes nuts and seeds; dairy; eggs; flesh food;, dark green leaves; other provitamin A-rich fruits and vegetables; and other fruits and vegetables.[14] For each food group, women consuming at least one time in the previous week were assigned a score of 1 and those who had not consumed were assigned a score of 0. A total diet diversity score was then computed representing the sum of these food groups (range from 0 to 9), and treated as a categorical variable (< 4, 4–7, > = 8). Household food security was accessed using FANTA’s Household Food Insecurity Access Scale and categorized into food secure, mild, moderate and severe food insecurity categories. [15]

Frequency distributions were calculated for (i) past history of dental disease, (ii) use of different oral hygiene products, (iii) access to dental treatment, and (iv) types of treatment received. Chi square statistics were applied to test the associations between distributions of risk factors and the presence of missing and damaged teeth. The risks of having a missing or damaged tooth by different maternal and household factors were calculated as odds ratios (OR) with 95% confidence intervals, unadjusted and adjusted to generate a robust standard error that accounted for clustering in the study design. The variables that were hypothesized to be associated with missing and damaged teeth included age, education level, occupation, caste, body mass index, diet diversity and wealth index. All analyses were conducted on the quantitative data analysis software StataCorp 15.1.

Ethical approval was obtained from the Institutional Review Boards of Johns Hopkins Bloomberg School of Public Health (IRB number: 4937)and on an annual basis from the Nepal Health Research Council, an autonomous body under the Ministry of Health, Nepal, (Reg. No.: 91/2016). Heads of households, newly married women and mothers or caregivers of children < 5 provided signed or verbal informed consent, depending on the level of education.

## Results

**Fig 1:**
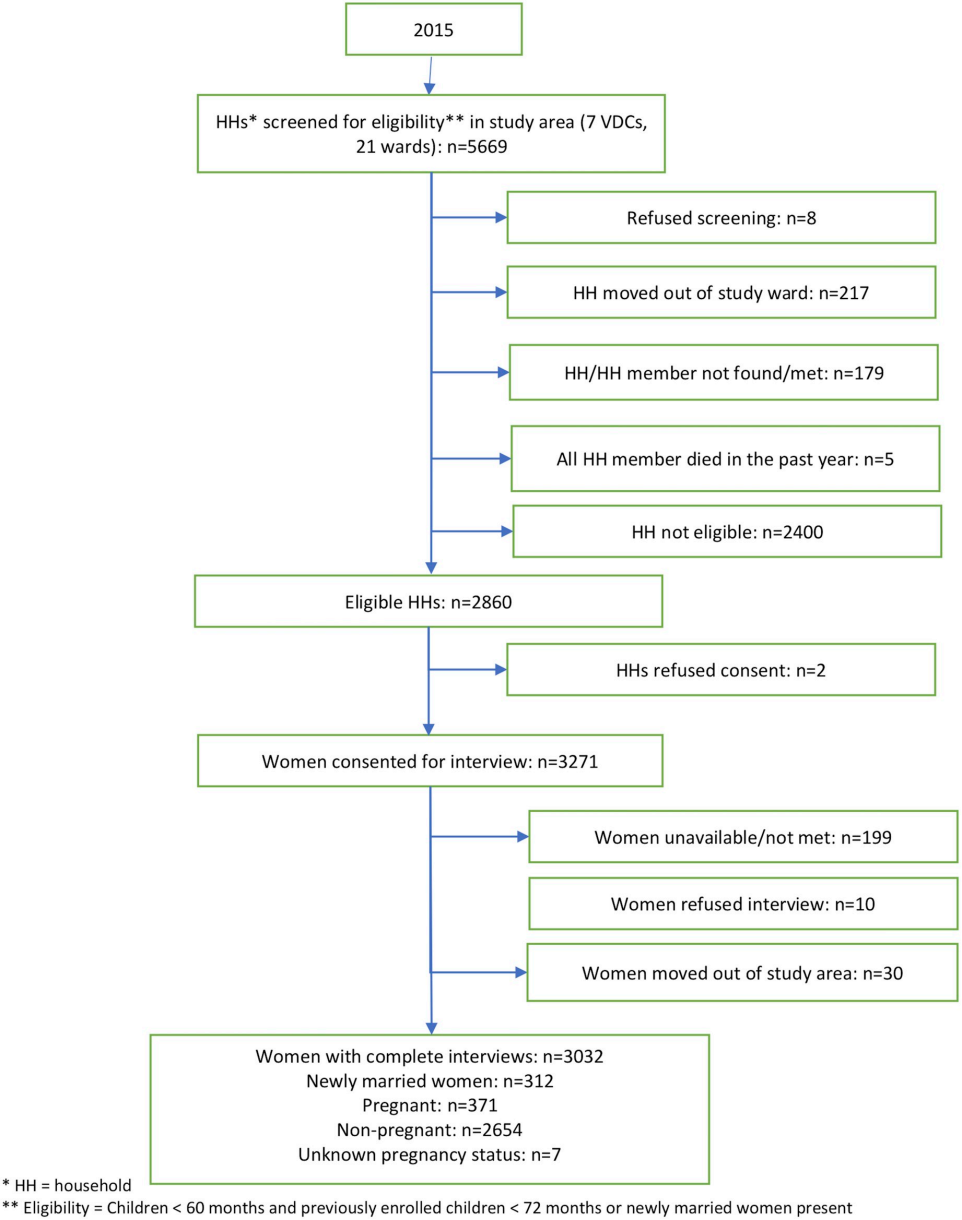
Consort diagram for the analytical sample of women of reproductive age in the Tarai.

Based on a cluster sampling design, 2,860 households were eligible for inclusion in the study. Of a total of 3,274 women eligible for interview, 3,007 (91.8%) women completed the oral health self-reported assessment modules after excluding women who were unavailable for interview (n = 199), refused interview (n = 10), moved out of the study area (n = 30) or were 50 years and older grandmother caretakers (n = 25). (Fig 1)

### Women’s characteristics

Mothers’ and newlywed women’s ages ranged from 14 to 49; 62.3% were 20 to 29 years of age and 3.3% were 40 to 49 years of age. More than three-quarters of households (82.5%) were food secure and 1.9% of the households were classified as severely food insecure. Most women (81.5%) brushed their teeth at least once a day, and 16.8% reported brushing twice a day or more frequently. Toothbrush and tooth paste were the most common tooth cleaning supplies used. Some women (1.1%) also used toothpicks or dental floss to clean interdental surfaces of the teeth. (Table 1)

**Table 1:** Socio-demographic characteristics and dental practices of women of reproductive age in Tarai, Nepal, 2015.

	n, %		
Characteristics	Total (3000, 100%)	Missing or damaged teeth (688, 22.9%)	Normal teeth (2319, 77.1%)
**Age (years)**^2^			
< 20	372 (12.3)	48 (6.8)	324 (13.9)
20–29	1873 (61.8)	398 (56.3)	1475 (63.4)
30–39	662 (21.8)	208 (29.4)	454 (19.5)
40–49	100 (3.3)	34 (4.94)	66 (2.85)
**Education (years of school)**^3^			
None	1,619 (53.8)	348 (50.6)	1,271 (54.8)
Any grade	1,388 (46.2)	340 (49.4)	1,048 (45.2)
**Occupation**^1^^,^^3^			
Student/ Non-earning	2,486 (82.8)	549 (79.9)	1,937 (83.7)
Wage employment	163 (5.4)	42 (6.1)	121 (5.2)
Business/ trade/self	107 (3.6)	21 (3.1)	86 (3.7)
Salaried worker	39 (1.3)	10 (1.5)	29 (1.3)
Homestead	207 (6.9)	65 (9.5)	141 (6.1)
**Caste**^2^			
Brahmin/ Chettri	290 (9.7)	96 (14.1)	194 (8.4)
Dalit/ Janajati	784 (26.3)	171 (25.2)	613 (26.6)
Others	1,601 (53.7)	325 (49.3)	1,266 (55.0)
Newars	39 (1.3)	10 (1.5)	29 (1.3)
Muslims	267 (8.9)	67 (9.9)	200 (8.7)
**Wealth Index**			
< 0	1,670 (55.5)	366 (53.2)	1,304 (56.2)
> = 0	1,337 (44.5)	322 (46.8)	1,015 (43.8)
**Diet**			
Diet diversity score < 4	70 (2.3)	15 (2.2)	55 (2.4)
Diet diversity score 4–7	2,330 (77.5)	533 (77.5)	1,797 (77.5)
Diet diversity score > = 8	607 (20.2)	140 (20.4)	467 (20.1)
**Household food insecurity**^2^			
None	2,480 (82.5)	544 (79.1)	1,936 (83.5)
Mild	291 (9.7)	71 (10.3)	220 (9.5)
Moderate	178 (5.9)	60 (8.7)	118 (5.1)
Severe	58 (1.9)	13 (1.9)	45 (1.9)
**Cleaning/ brushing teeth**			
Once daily	2,450 (81.5)	537 (78.1)	1,913 (82.5)
> = 2 times per day	505 (16.8)	137 (19.9)	368 (15.9)
1–6 times per week	50 (1.7)	14 (2.0)	36 (1.6)
**Tooth cleaning supplies**			
Toothpaste	2,368 (78.8)	567 (82.4)	1,801 (77.7)
Toothbrush	2,303 (76.6)	544 (79.1)	1,759 (75.8)
Bamboo stick (neem pata)	1,104 (36.7)	243 (35.6)	861 (37.2)
Charcoal	103 (3.4)	24 (3.5)	79 (3.4)
Toothpick/ dental floss	33 (1.1)	33 (1.1)	25 (1.1)

^1^ Non-earning occupation includes housewife and Female Community Health Volunteer, a position that has no associated salary; Homestead occupation includes agriculture, livestock, poultry, and aquaculture

On chi square test,

^2^ statistically significant at p value of <0.001;

^3^ statistically significant at pvalue of 0.01

### Prevalence of missing and damaged teeth

Nearly a quarter of women (22.9%; 95% CI: 21.4–24.4) had one or more missing or damaged tooth in their oral cavity. (Table 2) Among women 30–39 years of age, 19.3% had one or more missing teeth and 18.7% had one or more damaged teeth. Some participants reported having difficulty orienting themselves with the pictorial diagram of the oral cavity, which can be a limitation since participants use the simulation to indicate missing or damaged teeth in a particular sextant of the mouth.

**Table 2:** Prevalence of missing or damaged teeth among women in Tarai, Nepal, 2015.

Age group (years)	Total	Missing teeth ^1^			Damaged teeth^2^			Missing or Damaged teeth
		Any	1 tooth	> = 2 teeth	Any	1 tooth	> = 2 teeth	
<20	372	14 (3.8)	14 (3.8)	0	37 (9.9)	33 (8.9)	4 (1.1)	48 (12.9)
20–29	1873	204 (10.9)	187 (9.9)	17 (0.9)	252 (13.5)	209 (11.2)	43 (2.3)	398 (21.3)
30–39	662	128 (19.3)	104 (15.7)	24 (3.6)	124 (18.7)	99 (14.9)	25 (3.8)	208 (31.4)
40–49	100	25 (25.0)	19 (19.0)	6 (6.0)	18 (18.0)	14 (14.0)	4 (4.0)	34 (34.0)
Total	3007	371 (12.3)	324 (10.8)	47 (1.6)	431(14.3)	355 (11.9)	76 (2.5)	688 (22.9)

^1^ Missing teeth are those that were extracted due to a dental problem or those that shed due to looseness or age. Third molars were considered missing only if the respondent reported it being erupted and then lost for any reason. If the third molar did not erupt at all, it was not recorded as missing.

^2^ Damaged teeth were defined as those that were decayed, cracked, chipped, broken, or mottled.

* All values are row percentages

In multivariable adjusted models, the relative odds of having a missing or damaged tooth rose monotonically with increasing age, with women 30 to 39 years of age at a 4.08 (95% CI 2.32–7.16) times higher odds of having missing or damaged teeth than the referent group, women under 20 years of age. (Table 3) Relative to “higher” Brahmin or Chettri castes, women from Dalit and Janajati castes had a lower odds of having missing or damaged teeth (OR 0.65, 95% CI 0.46–0.91). (Table 3) Women with no formal education had 24% (95% CI 4–39) lower odds of having missing or damaged teeth that those with any formal education. There were no differences in the relative odds of having teeth missing or damaged by occupation, nutritional indicators (BMI, diet diversity scores) or socioeconomic status (wealth index).

**Table 3:** Odds ratio (OR) of having missing or damaged teeth in women of reproductive age, Tarai region, Nepal, 2015.

Characteristics	Unadjusted OR		Adjusted OR^1^	
	OR	95% CI	OR	95% CI
**Age (years)**				
< 20	Ref	-	-	-
20–29	1.82	1.32–2.52	1.83	1.27–2.63
30–39	3.09	2.19–4.36	3.14	2.12–4.68
40–49	3.48	2.08–5.81	4.08	2.32–7.16
**Education (years of school)**^3^				
None	0.84	0.71–1.00	0.76	0.61–0.96
Any grade	Ref	-	-	-
**Occupation**^2^				
Student/ Non-earning	Ref	-	-	-
Wage employment	1.22	0.86–1.76	1.12	0.75–1.68
Business/ trade/self	0.86	0.53–1.40	0.78	0.46–1.29
Salaried worker	1.21	0.59–2.51	1.22	0.56–2.64
Homestead	1.62	1.19–2.19	1.26	0.90–1.76
**Caste**^3^				
Brahmin/ Chettri	Ref	-	-	-
Dalit/ Janajati	0.56	0.42–0.76	0.65	0.46–0.91
Others	0.53	0.41–0.70	0.63	0.46–0.87
Newars	0.69	0.32–1.49	0.66	0.28–1.53
Muslims	0.68	0.46–0.98	0.77	0.50–1.18
**Body mass index**^4^				
<18.5	1.18	0.97–1.46	1.09	0.89–1.36
18.5–24.9	Ref	-	-	-
25–29.9	1.44	1.04–2.00	1.09	0.76–1.55
> = 30	0.83	0.33–2.03	0.57	0.23–1.45
**Diet**				
Diet diversity score < 4	0.91	0.49–1.66	1.09	0.56–2.12
Diet diversity score 4–7	0.97	0.80–1.22	1.01	0.80–1.28
Diet diversity score > = 8	Ref	-	-	-
**Wealth Index**				
< 0	0.88	0.74–1.04	1.04	0.84–1.29
> = 0	Ref	-	-	-

^1^ Odds ratios adjusted for age, level of education, occupation, caste, BMI, MUAC, wealth index and diet.

^2^ Missing values– 5 (0.2%);

Non-earning occupation includes housewives and female community health volunteers; Homestead occupation includes agriculture, livestock, poultry, aquaculture

^3^ Missing values– 26 (0.86%)

^4^ measured in weight in kg/height in m^2^

### Past history of dental pain and access to treatment

Over a recall period of 6 months, 528 (17.6%) women reported having pain or discomfort in their oral cavity. Tooth decay, swelling of the gums (also known as gingivitis), drinking cold water or eating sweet foods were among the main reasons for intermittent or constant pain for 53.6% of women who reported pain or discomfort. Swelling of the gums (24.4%) and injury to the mouth (16.1%) were among the other common reasons. Of women who had pain or discomfort, only 53.8% sought treatment, with the most prevalent care providers being dentists (41.9%) and pharmacists (local shop compounders)/village doctors (40.5%).

Pharmacies (37.7%) were the most common treatment facilities visited for pain and discomfort, followed by dental clinics (31.3%), hospitals (11.9%) and primary health care centers (6.7%). Most women (79.9%) were given medications for pain relief, reportedly to fight infections or treat fever. Extraction, tooth cleaning and restorations were some of the other treatment options that were provided. Approximately 9.5% used home remedies such as salt-water gargles, compression with oil of cloves, balm, or roasted ginger at the site of pain to get relief. (Table 4)

**Table 4:** History of pain or discomfort in the mouth and corresponding treatment over a 6 month period among women of reproductive age, Tarai, Nepal, 2015.

Characteristics	Counts (N = 3007)	Frequency (%)
History of pain/ discomfort^1^ (Yes)	528	17.6
**Reasons for pain/ discomfort (n = 528)**^4^		
Decay	283	53.6
Swelling of gums	129	24.4
Injury to mouth	85	16.1
Others	59	11.2
Loose teeth	18	3.4
Broken or cracked teeth	11	2.1
Don’t know	2	0.4
**Treatment provider (n = 284)**^3^		
Pharmacist/ village doctor	115	40.5
Doctor/ Dentist	119	41.9
Health worker^2^	23	8.1
Relative/ friend	14	4.9
Traditional healer/ Shaman	13	4.5
**Treatment facility (n = 284)**		
Pharmacy	107	37.7
Dental clinic	89	31.3
Hospital	32	11.3
Primary health care center	19	6.7
Home	18	6.3
Other’s home	14	4.9
Others	5	1.8
**Treatment received (n = 284)**^4^		
Medicine	227	79.9
Extraction	60	21.1
Tooth cleaning	42	14.8
Home remedy	27	9.5
Filling (restoration)	23	8.1
Others	11	3.8
No treatment / don’t know	5	1.8

^1^ Pain/ discomfort in mouth includes history of tooth ache, currently or in the past 6 months

^2^ Includes government health worker (18), female community health volunteer (1) and non-governmental organization health worker

^3^Among the 528 women who had had pain or discomfort in their teeth in the past 6 months, 284 women sought treatment.

^4^ Multiple responses (upto 3) from women to questions on reasons for pain/discomfort and treatment received, thus the ns’ done add to 528 (women who had pain/discomfort in the last 6 months) and 284 (women who received treatment) respectively

History of pain/discomfort history was significantly lower (36.6%, p value—< 0.001) among those who did not report a missing or damaged tooth compared to those with history of missing or damaged teeth.

## Discussion

This study provides an initial estimate of the prevalence of missing or damaged teeth among women of reproductive age across the southern, low lying plains (the *Tarai*) of Nepal, employing a interviewer-administered tooth assessment module. By this method, 12.3% of surveyed women reported tooth loss, 14.3% reported damaged teeth, and 22.8% reported either condition. More than 80% of the adult women reported practicing oral hygiene on a daily basis. Additionally, women 40–49 years of age and those from the Brahmin and Chhetri castes had higher odds of reporting a missing or a damaged tooth.

Our findings that nearly a quarter of women had lost or broken teeth is consistent with findings among adults in other Asian populations, including Indian, Singapore and China which have generated prevalence estimates of dental caries and periodontitis ranging from 28% to 34%. [16–18] These conditions are among the most common oral diseases along with cancers of the mouth and contribute to the burden of missing or damaged teeth in populations. [19–21] The high prevalence of oral diseases is usually attributed diverse dietary factors such as consumption of sugary drinks and intake of sticky food, water fluoride levels, oral hygiene, access to (fluoridated) tooth cleaning products, and genetic factors. [22, 23]

Most adult women reported practicing oral hygiene measures such as tooth brushing on a regular basis, as has been reported previously for Nepalese adults. [24] The use of tooth brushes and fluoridated dentrifices is more prevalent in the Tarai region than other ecological belts of the country.[24] Toothbrush and tooth paste were most commonly used oral dentifrices. Bamboo or neem twigs and charcoal were also supplemental household products used for tooth brushing. Another study conducted in the Tarai region of Nepal also cited similar use of products in addition to mud, dirt and ash to clean teeth, which are abrasive in nature and can lead to teeth damage. [25] Past studies have shown that even though laypersons practice regular oral hygiene, they are not aware of fluoridated dentrifices, which reduce the risk of dental decay pertaining to a chemical change in the tooth enamel structure. [25–27] In this study, we were unable to capture the fluoridation status of locally available dentrifices. The inverse odds of damaged or missing teeth by caste and education status as seen in this study could be mediated by a propensity to consume a more cariogenic diet in higher socioeconomic groups. [28–30]

The data on treatment history showed that women in rural Nepal had access to treatment options—both diagnostic and therapeutic. Medications were the most common treatment received. Restorative treatments such as fillings and artificial prosthetics were far less common than extractions. It is important to note, however, that while our study did not evaluate the quality of the treatment services to which respondents were referred, limited financial resources and health care infrastructure, and long distance to facilities with advanced dental care services in most rural areas would suggest most services provided in the Tarai are quite basic compared to those available in urban centers. [31–33] When services are sought, a recent study conducted among pregnant women in rural Nepal revealed little awareness of preventive dental services, showing that treatment seeking behavior remains driven by pain or discomfort in the oral cavity. [25]

This study is among the few to have gathered data on the prevalence of damaged and missing teeth and treatment sought for such conditions by women in rural populations, adding new knowledge to a limited evidence base. The use of a tooth assessment module with minimal training of data enumerators enabled the collection of information needed to generate these estimates. The tool can be scaled in Nepal and adapted in other countries in the region employing similar cadres of community survey workers to collect information on oral health. [34] However, it is important to note that damaged and missing teeth are only a subset of a host of oral diseases that can be present and, thus, do not represent the total burden or extent of oral diseases in Nepal. An interviewer-administered assessment module, such as the one used in this study, has the potential to reveal the burden, however, of pain and damage in the oral cavity, guide the roll out of more sophisticated point-of-care diagnostics in low-resource environments, be applied in a standardized way for oral health assessments and provide sufficient information for basic oral health education. A limitation of this tool is that we were not able to collect information on restorative treatment, a proxy to dental disease history, normally collected via the DMFT index or via oral radiographs, as the recognition of restorative materials (metallic or tooth colored) was not included in the training for data enumerators.

Another limitation to the study is the self-reported method of enumerating life time history of missing and damaged teeth, which is subject to response and recall bias, and selection bias due to the very specific inclusion criteria. Shyness, fear of stigmatization as well as lack of awareness about oral health issues may have led to discrepancies. While the study has generated novel findings, future studies should cross-validate findings from this simplified assessment tool with methods such as the DMFT index to evaluate its use as an inexpensive method for estimating the prevalence of lost, broken, carious and dysfunctional dentition in low resource settings lacking dental services and trained dental researchers. [6] The assessment tool can further be expanded to estimate the prevalence of missing and damaged teeth and oral hygiene practices in other regions of the country.

In conclusion, a primary objective of this assessment was the application of a simple survey tool to estimate the extent and severity of missing and damaged teeth in an adult female population. Our module was readily utilized by trained, lay data collectors and health care workers allowing data on clinically important oral health conditions to be collected. The findings suggest that missing and damaged teeth represent a major burden among women of reproductive age in the southern plains of Nepal.

## Supporting information

S1 Fig Randomly selected village development committees in the Tarai region of Nepal, 2015. (PDF)

S2 Fig Simulation of the oral cavity used for training field staff and to aid in data collection. (PDF)

S1 File The tooth assessment module. (PDF)
