# Correction: Prevalence of damaged and missing teeth among women in the southern plains of Nepal: Findings of a simplified assessment tool

**DOI:** 10.1371/journal.pone.0228401

**Published:** 2020-01-24

**Authors:** Priyanka Agrawal, Swetha Manohar, Andrew L. Thorne-Lyman, K. C. Angela, Binod Shrestha, Rolf D. Klemm, Keith P. West

There are errors in the author affiliations. Affiliation #2 incorrectly appears as affiliation #3 and #6. The correct affiliations are as follows:

Priyanka Agrawal^1^, Swetha Manohar^2^, Andrew L. Thorne-Lyman^2,3^, K. C. Angela^2^, Binod Shrestha^4^, Rolf D. Klemm^2,5^, Keith P. West^2^

**1** International Injury Research Unit, Department of International Health, Johns Hopkins Bloomberg School of Public Health, Baltimore, Maryland, United States of America, **2** Center for Human Nutrition, Department of International Health, Johns Hopkins Bloomberg School of Public Health, Baltimore, Maryland, United States of America, **3** Department of Nutrition, Harvard T.H. Chan School of Public Health, Boston, Massachusetts, United States of America, **4** PoSHAN Study Team, Johns Hopkins University, Kathmandu, Nepal, **5** Helen Keller International, New York, New York, United States of America.

There is a typographical error in the “Setting” subsection of the Abstract. The correct sentence is: 21 wards in seven Village Development Committees across the Tarai of Nepal in 2015.

References 1 and 2 are incorrectly placed within the second sentence of the first paragraph of the Introduction. The second sentence and references 1 and 2 should appear as: Reliable prevalence estimates, with consequent quality of life and economic burdens, of untreated dental diseases, remain virtually absent to date for lack of technical capabilities in resource poor settings. [1, 2]

A full stop is missing at the end of the fifth sentence of the first paragraph of the Introduction. The correct sentence is: Notwithstanding the paucity of data, estimates that do exist suggest poor dental health to be a global public health problem, with 3.5 billion people having treatable, morbid dental conditions [5].

Reference 6 is incorrectly placed within the second sentence of the second paragraph of the Introduction. The correct sentence is: Commonly dental disease prevalence measures and indices such as the World Health Organization’s (WHO) recommended “decayed, missing, filled teeth” index (DMFT), the Oral Hygiene Index—Simplified (OHI-S), and the Community Periodontal Index for Treatment Needs (CPITN) continue to remain under the sole purview of dental health scientists, clinicians and technical specialists, although structured questionnaires have occasionally been used to gather information on dental decay and pain by trained dentists in school and village settings. [1, 2, 6–8].

A full stop is missing at the end of the fourth sentence in the second paragraph of the Introduction. The correct sentence is: Specifically, while generating estimates of the burdens of most severe oral diseases, the GBD fails to capture antecedent conditions to severe periodontitis such as early tooth decay or poor oral hygiene practices that precede clinical disease [5].
