# Supplementary material for: Prevalence of damaged and missing teeth among women in the southern plains of Nepal: Findings of a simplified assessment tool
Source: PLoS One. 2019 Dec 3;14(12):e0225192. doi: 10.1371/journal.pone.0225192 (PMC6890177; doi:10.1371/journal.pone.0225192)

S2 Figure: Simulation of the oral cavity used for training field staff and to aid in data collection

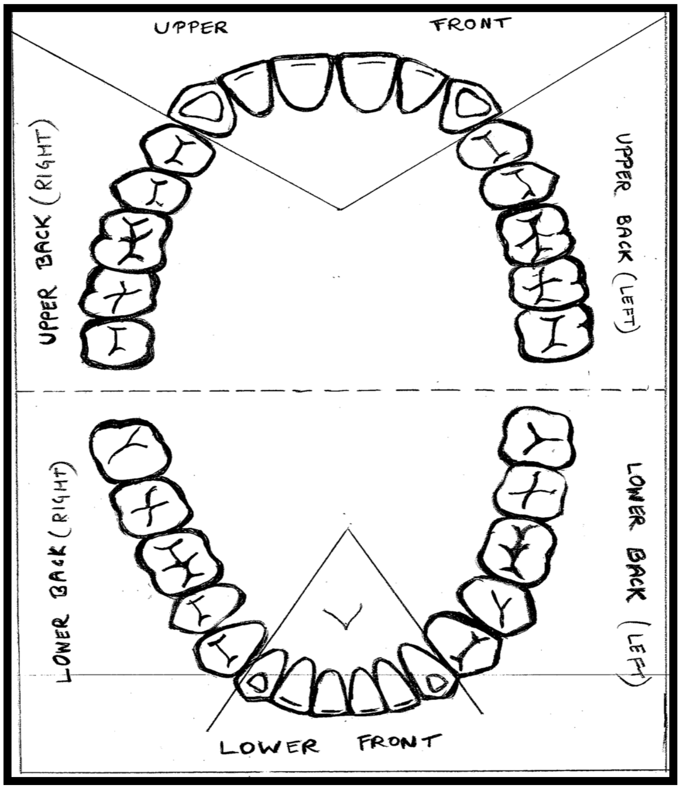

Supplement: S2 Fig — (PDF) [file pone.0225192.s002.pdf]
