# Supplementary material for: Prevalence of damaged and missing teeth among women in the southern plains of Nepal: Findings of a simplified assessment tool
Source: PLoS One. 2019 Dec 3;14(12):e0225192. doi: 10.1371/journal.pone.0225192 (PMC6890177; doi:10.1371/journal.pone.0225192)
Supplement: S1 File — (PDF) [file pone.0225192.s003.pdf]

S1 File: The tooth assessment module

Section O: Women's tooth assessment

Read aloud: I will now ask you some questions about your dental health and habits.

**1**	Have you ever had any problems in chewing food?	☐			0 = No → *If No or Don't know*, then skip to Q.2<br>1= Yes<br>9= Don't know
**1a**	***If YES***, what is the problem you have in chewing food?	☐			0 = Pain in the teeth<br>1 = Missing teeth<br>2 = Loose teeth<br>8 = Others<br>9 = Don't know
**2**	Do you brush your teeth?	☐			0 = No → *if NO*, then skip to Q.3<br>1= Yes
**2a**	***If YES***, how many times do you brush your teeth?	☐			0= Rarely (less than 1 time per week)<br>1= Sometimes (1-6 times per week)<br>2= Once daily<br>3= 2 or more times a day
**2b**	What do you use to brush your teeth?<br>*(Please mark all answers)*				0= No<br>1=Yes
**A**	Tooth brush	☐	**D**	Charcoal/Ash	☐
**B**	Tooth paste	☐	**E**	Tooth pick	☐
**C**	Tree twigs	☐	**F**	Dental floss	☐
**3**	Have you had pain/discomfort in your mouth in the last 6 months?<br>*(If yes, please probe)*	☐			0= No → ***If NO,*** skip to Q.4<br>1 = Yes, and still have pain<br>2 = Yes, but no longer have pain<br>9 = Don't know → ***If Don't know,*** skip to Q.4
**3a**	***If YES***, do you know why you had the pain/discomfort?<br>(Code *up to 2 answers)*	3aa ☐<br>3ab ☐			0= decay<br>1= loose teeth<br>2= broken or cracked teeth after biting on something hard<br>3= injury to mouth<br>4= swelling of gums<br>8= other reason<br>9= don't know
**3b**	From whom did you seek treatment for the pain/discomfort?	☐			0= did not seek treatment→Skip to Q4<br>1= relative/friend/self<br>2= traditional healer/Shaman<br>3= FCHV<br>4= other government health worker<br>5= NGO health worker<br>6= pharmacist/village doctor<br>7= doctor/dentist<br>9=don't know
**3c**	Where did you seek treatment for the pain/discomfort?	☐			0= home<br>1= other's home<br>2= pharmacy<br>3= dental camp<br>4= PHC/Health post/Sub health post/ outreach health facility<br>5= dental clinic<br>6= hospital<br>8= others<br>9= don't know
**3d**	What treatment did you receive for the pain/discomfort?<br>*(Code up to 3 answers)*	3da. ☐<br>3db. ☐			0= no treatment<br>1= home remedy<br>2= medicine<br>3= extraction<br>4= filling<br>5= tooth cleaning<br>8= others<br>9= don't know

				3dc.			
4	Do you have any missing teeth at the moment?					0= No →<br>1= Yes<br>9= Don't know	***If NO or Don't know,***<br>skip to Q.5
4a	***If YES***, how many teeth are missing?					0-6 (record number of teeth missing in the boxes below)<br>9= Don't know	
		Back-Right		**Front**		Back-Left	
**4b**	Upper Jaw	4ba		4bb		4bc	
**4c**	Lower Jaw	4ca		4cb		4cc	
**5**	Do you have any damaged teeth at the moment?					0= No →<br>1= Yes<br>9= Don't know	***If No or Don't know,***<br>skip to end
**5a**	***If YES***, how many teeth are damaged?					0-6 (record number of teeth damaged in the boxes below)<br>9= Don't know	
		Back-Right		**Front**		Back-Left	
**5b**	Upper Jaw	5ba		5bb		5bc	
**5c**	Lower Jaw	5ca		5cb		5cc	
